# Mesothelial Cyst in the Uterine Myometrium: A Rare Localization

**DOI:** 10.7759/cureus.75158

**Published:** 2024-12-05

**Authors:** Gözde Arslan, Yeliz Acar Sabir

**Affiliations:** 1 Department of Pathology, Kastamonu Training and Research Hospital, Kastamonu, TUR; 2 Department of Obstetrics and Gynecology, Kastamonu Training and Research Hospital, Kastamonu, TUR

**Keywords:** benign cystic mesothelioma, degenerated leiomyoma, mesothelial cyst, myometrium, uterus

## Abstract

Mesothelial cysts in the uterus are exceedingly rare. A 41-year-old patient presented with complaints of abdominal pain, and transvaginal ultrasonography revealed an enlarged uterus with a hypoechoic intramural cystic mass measuring 7.2 × 3.3 × 3.6 cm in the posterior uterine corpus. Suspecting a degenerated leiomyoma, the patient underwent a total laparoscopic hysterectomy and bilateral salpingectomy. Intraoperatively, a cystic lesion approximately 7 cm in size was observed within the myometrium of the posterior uterine wall. Pathological examination, supported by immunohistochemical findings, confirmed the diagnosis of a mesothelial cyst. Mesothelial cysts can mimic various clinical and radiological entities, and definitive diagnosis is typically established through pathological evaluation following complete excision of the lesion.

## Introduction

Uterine mesothelial cysts are extremely rare. Intra-abdominal mesothelial cysts were first described in 1979 [1], and approximately 150 cases have been reported in the literature to date. To the best of our knowledge, only five of these cases are associated with the uterus, and only one published in 2019, as in our case, developed within the myometrium [2,3]. The factors triggering the growth of mesothelial cysts remain unknown, although developmental anomalies are often suspected. A history of abdominal surgery, pelvic inflammation, endometriosis, or mesothelial cysts of the round ligament are considered potential associations [4]. Herein, we present a case of a uterine mesothelial cyst that was surgically treated under the preoperative diagnosis of a degenerated leiomyoma.

## Case presentation

**Figure 1:**
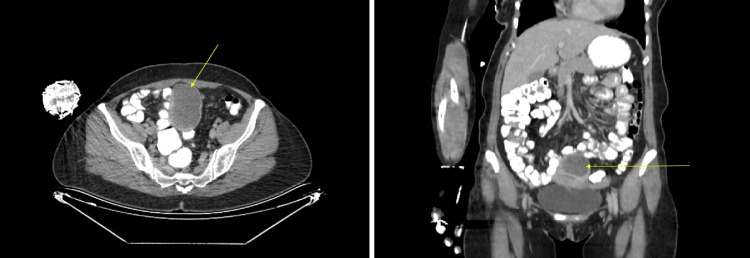
Computed tomography revealed a hypodense space-occupying lesion measuring 7.2 × 3.3 × 3.6 cm in the posterior wall of the uterine corpus (yellow arrow).

A 41-year-old female patient presented to the gynecology clinic with complaints of abdominal pain. Her medical history revealed two pregnancies, one ending in cesarean delivery years ago and the other in curettage, with no additional comorbidities or surgical history. Transvaginal ultrasonography demonstrated an enlarged uterus with a hypoechoic intramural cystic mass measuring 7.2 × 3.3 × 3.6 cm in the posterior uterine corpus. The endometrium appeared thin and regular, and the bilateral adnexal regions were unremarkable. Computed tomography further identified the lesion as a hypodense mass in the uterine corpus measuring 7.2 × 3.3 × 3.6 cm (Figure 1).

**Figure 2:**
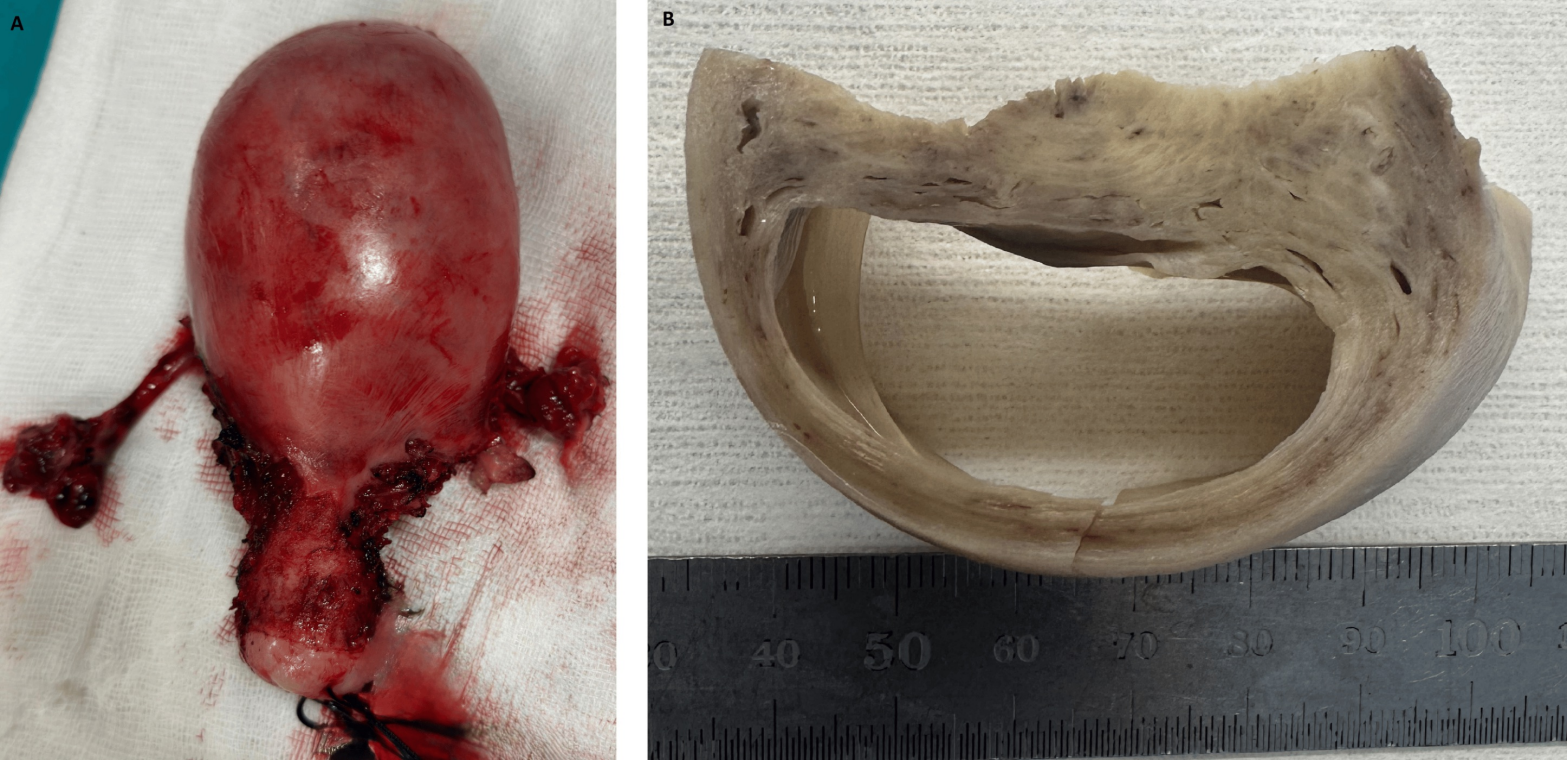
(A) The cyst in the posterior wall of the uterine corpus with an enlarged uterus. (B) The macroscopic appearance of the cyst within the myometrium, unrelated to the endometrium (top) and containing a septum on the left edge.

Under the preliminary diagnosis of a degenerated leiomyoma, the patient underwent total laparoscopic hysterectomy and bilateral salpingectomy. Macroscopic examination of the uterus revealed a 6 cm cystic lesion containing serous fluid within the posterior uterine wall (Figure 2).

**Figure 3:**
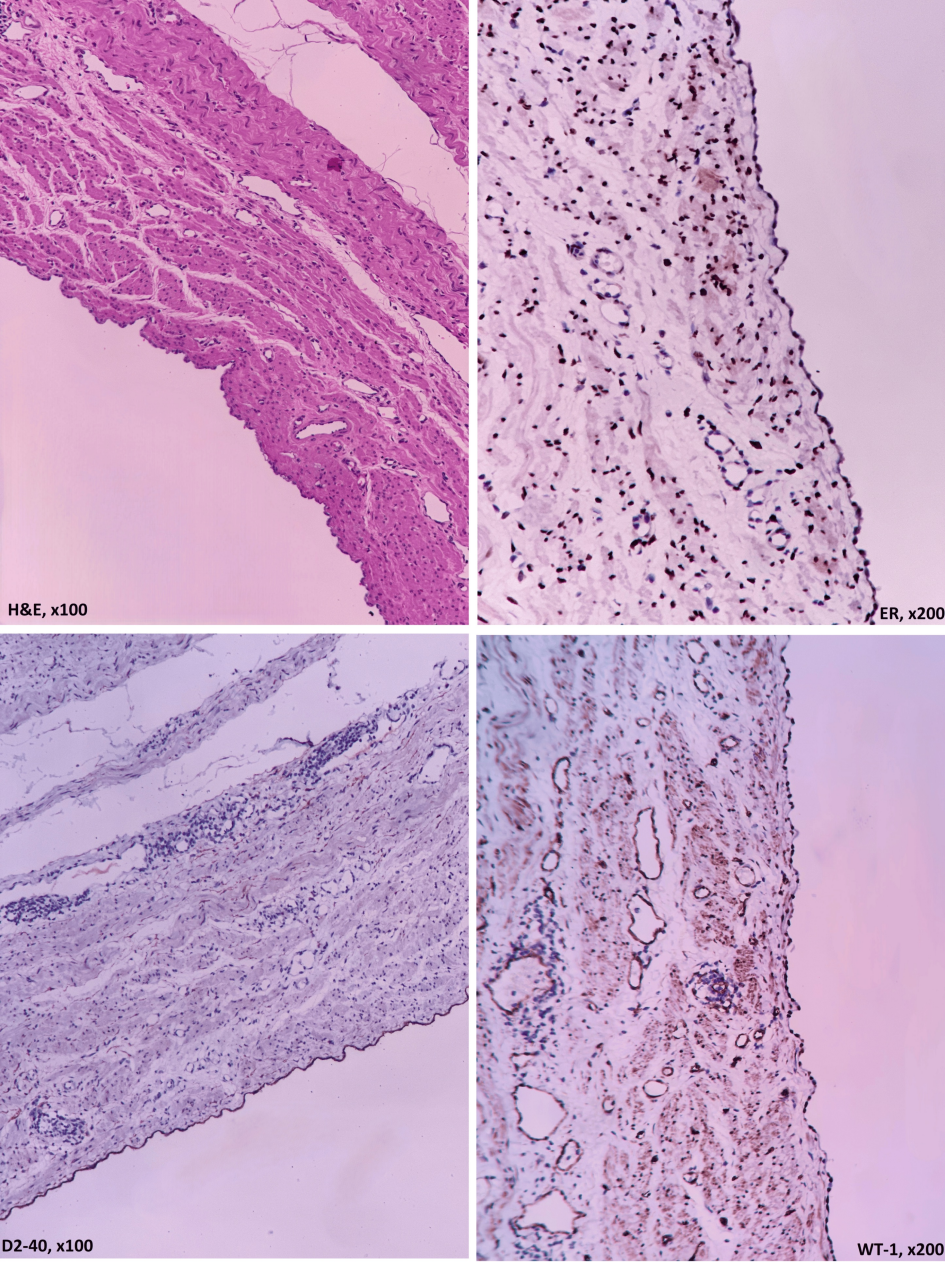
The cyst wall lined with a single layer of cuboidal mesothelial cells (H&E, ×100), and the expression of ER (×200), D2-40 (×100), and WT-1 (×200) in the cells lining the cyst wall. H&E: hematoxylin and eosin

Upon incision, no solid or papillary structures were observed on the cyst wall, although septation was noted in one area. Microscopic evaluation revealed a cystic lesion lined with a single layer of benign cuboidal mesothelial cells located within the myometrium, unrelated to the endometrium or serosa. Immunohistochemical studies showed positive staining of the cyst epithelium with calretinin, D2-40, WT-1, ER, and PR (Figure 3).

Extensive sampling was performed to exclude degenerated leiomyoma, but no areas indicative of leiomyoma were identified. The case was diagnosed as a uterine mesothelial cyst based on the histomorphological and immunohistochemical findings. No pathology was observed during the patient's follow-up.

## Discussion

Since 1985, no cases of mesothelial cysts in the uterine myometrium have been reported in the literature, except for one case in 2019, which, similar to our case, involved a mesothelial cyst developing within the myometrium [3]. Of the 150 cases of mesothelial cysts reported in the literature, only five were uterine-related. One was described as a multicystic lesion attached to the serosa by a pedicle between the left uterine fundus and ovary [5], another presented as multiple small cysts on the serosal surface of the uterine corpus [4], one resembled our case within the myometrium [3], and the remaining two were reported as lesions adhering to the myometrium on the posterior uterine wall [2,6].

Based on the current literature, our case represents the second instance of a mesothelial cyst developing within the myometrium. Due to their prevalence in women of reproductive age, some studies suggest a potential relationship between sex hormones and mesothelial cysts. However, since mesothelial cysts of the round ligament do not demonstrate immunohistochemical expression of ER or PR, some authors propose that these lesions are independent of sex hormones [4,5,7]. In the five uterine mesothelial cyst cases, the patients were aged 27-47 years, but the menopause status of women in their 40s is unknown, and while ER and PR expressions were negative in one of the five articles, immunohistochemical studies related to ER and PR were not reported in the other four articles [2].

The developmental process of these cysts remains controversial due to the limited number of reported cases and the need for further research. Mesothelial cysts share similar morphological and histopathological features with benign cystic mesothelioma [7]. Both can manifest as inclusion cysts within the pelvic cavity. While mesothelial cysts are typically solitary, unilocular, and benign, with epithelia composed of well-differentiated mesothelial cells, benign cystic mesothelioma is often multilocular and considered a reactive lesion or a neoplasm with low malignant potential and a tendency for recurrence [3,4]. Uterine mesothelial cysts lack specific clinical features. They may be asymptomatic or present with a palpable abdominal mass or pelvic pain in some patients. Radiological diagnosis is challenging due to their similarities with leiomyomas exhibiting cystic degeneration, and they may be misdiagnosed preoperatively as degenerated leiomyomas, as in this case [2]. The characteristic microscopic appearance of a single layer of cuboidal mesothelial cells lining the cyst wall, along with positive staining for specific mesothelial markers such as calretinin, WT-1, mesothelin, and HBME, aids in the diagnosis of pelvic mesothelial cysts [8-10]. Estrogen and progesterone expression remain variable, and no definitive conclusions have been reached [4]. Although most authors describe mesothelial cysts as benign lesions with low recurrence and malignant potential, cases with recurrence rates of up to 27% in benign cystic mesotheliomas and malignant transformation have been documented in the literature [5,7]. Treatment options include cyst excision; however, the thin cyst walls often complicate complete excision and result in high recurrence rates. To minimize recurrence, total or partial hysterectomy is considered the optimal approach, especially for older patients or those who do not plan to have children [2].

## Conclusions

In conclusion, mesothelial cysts are extremely rare lesions in the uterus, and definitive diagnosis is typically established through pathological examination. Further case reports and advanced studies will help us better understand the development process of this lesion. Awareness of this uncommon lesion and its inclusion in the differential diagnosis of pelvic pain in women of reproductive age can facilitate patient management. Although complete excision of the cyst wall may be challenging, the prognosis is favorable, and hysterectomy, when indicated, significantly reduces the risk of recurrence.
